# Bloodstream infections in cancer patients in central India: pathogens and trends of antimicrobial resistance over a 5-year period

**DOI:** 10.1099/acmi.0.000673.v5

**Published:** 2024-10-29

**Authors:** Sonali Choudhari, Ruchita Gawande, Jerestin Watchmaker, Pooja Bamnote, Pradeep Mishra, Pankaj Dwivedi

**Affiliations:** 1 National Cancer Institute, M.S, Khasra No, 25, Outer Ring Rd, Mouza, Jamtha, Maharashtra 441108, India

**Keywords:** bloodstream infections, haematological malignancies, multidrug-resistant organisms

## Abstract

**Introduction.** Bloodstream infection (BSI) is a common complication with a high fatality rate in cancer patients. There are notable variations in the epidemiology of BSI over time and among different countries. Infections due to multidrug-resistant organisms (MDROs) such as extended-spectrum beta-lactamases (ESBLs) and carbapenem-resistant *Enterobacteriaceae* (CRE) are increasing. This may lead to inadequate empirical antibiotic therapy, increasing the antimicrobial resistance (AMR) problem and unfavourable outcomes in these immunocompromised patients. There is paucity of data pertaining to AMR in such vulnerable patients from developing countries such as India. The aim of this study was to investigate the distribution of the bacterial pathogens causing BSI and the AMR trend in cancer patients in central India.

**Methodology.** This single-centre retrospective observational study was conducted in a tertiary care cancer hospital. Patients with solid organ and haematological malignancies, both adults and paediatric, who had blood cultures sent to the microbiology laboratory from January 2018 to December 2022 were included. Blood cultures were processed using the BacT/ALERT 3D system (bioMérieux, France), and the identification of the bacteria and their antimicrobial susceptibility (AST) was performed using the Vitek 2 compact system (bioMérieux, France). Electronic medical records and microbiology lab records were used to retrieve the demographic and microbiological data. Microsoft Excel (RRID:SCR_016137) was used to enter and tabulate the data. Statistical analysis was performed using SPSS version 29 (RRID:SCR_002865).

**Results.** A total of 687 isolates from 524 patients were studied. Gram-negative bacteria (64%) were the commonest cause of BSI in the studied patients, followed by Gram-positive cocci (25%) and fungal isolates (9%). Ten cases were polymicrobial. *Escherichia coli* (*n*=140) was the most common among the isolated pathogens, followed by *Klebsiella* species (*n*=103), *Pseudomonas* species (*n*=102), and coagulase-negative staphylococci (CONS) (*n*=92). Among the 140 isolates of *E. coli*, 66% were extended-spectrum β-lactamase (ESBL) producers and 26% were resistant to carbapenem. Among the 103 isolated *Klebsiella* species, 50% were carbapenem resistant and 36% were ESBL producers. Among enterobacterales, the CRE rate was 34%. Carbapenem resistance was seen in 25% of *Pseudomonas* species and 53% of *Acinetobacter* species isolates. *Klebsiella* species were the most resistant pathogens isolated. CONS comprised 56% of all Gram-positive isolates, followed by *Staphylococcus aureus* (36%), enterococci species (11%), and streptococci species (3%). Methicillin resistance was 60% in CONS and 64% in *S. aureus*. One vancomycin-resistant enterococcus was isolated. Non-*albicans Candida* was the most common fungal pathogen. The sensitivity to fluconazole was 84% in non-*albicans Candida* species, while only one isolate of *Candida albicans* was resistant to fluconazole. The trend of pathogens was insignificant over 5 years, with Gram-negative bacteria being the commonest. Further, there was no significant change in the trend of ESBL and CRE resistance pattern over 5 years.

**Conclusion.** Gram-negative bacteria were the most common isolated pathogens from BSI with a higher antimicrobial resistance rate in cancer patients. The CRE rate of 34% is alarming, limiting the choices for empirical antibiotic therapy.

## Data Summary

All data associated with this work are reported within the article.

## Introduction

Cancer is a significant cause of morbidity and mortality; it was the top reason for deaths in 2018, with an estimated 18.1 million new cases and 9.6 million fatalities [1]. In the USA, infection-related mortality accounts for ~60% of deaths among patients with haematological malignancies and 50% of deaths among patients with solid organ tumours [2,3]. Bloodstream infection (BSI) is a frequent complication in patients with cancer [4]. In addition to being the main reason for hospitalization, BSI contributes to high fatality rates in this population [5].

Treatment with appropriate antibiotics at an early stage is critical for the efficient management of infections in cancer patients. Delaying antibiotic treatment increases the length of hospital stays and leads to unfavourable outcomes [6].

There are published guidelines and regional recommendations (such as the Sanford Guide to Antimicrobial Therapy, the Practice Guidelines of the Infectious Diseases Society of America, and the Treatment Guidelines for Antimicrobial Use in Common Syndromes, ICMR, India) for choosing the proper empirical antibiotics for infections. However, these recommendations should be reviewed frequently because the patterns of antimicrobial susceptibility and microbiological epidemiology are evolving constantly. The lack of timely access to local data on antibiotic usage is a primary cause for the failure of this upgradation, particularly in low-income countries [7].

Several key aspects should be evaluated before choosing empirical antibiotics for cancer patients considering the rise in antimicrobial resistance. This includes a predicted complicated clinical course according to a patient’s history of colonization or infection with resistant pathogens, the presence of additional risk factors for antibiotic resistance, the local epidemiology, and patterns of resistance in that hospital, unit, and location, and other patient-related factors (such as advanced age, comorbidities, localized infection, and shock) [8].

Over time, there are noticeable changes in trends in the epidemiology of BSI [9]. The prevalence of pathogens and their patterns of resistance differ greatly among countries, as well as among hospitals within a single country [10]. Infection with multidrug-resistant organisms (MDROs) presents an additional challenge due to limited therapeutic options [11].

Antimicrobial resistance (AMR) is a global problem. The WHO published a list of priority pathogens in 2017, highlighting the need for research and development for antibiotics for these resistant bacteria. The extended-spectrum beta-lactamase (ESBL) and carbapenem-resistant *Enterobacteriaceae* (CRE), carbapenem-resistant *Pseudomonas aeruginosa*, and carbapenem-resistant *Acinetobacter baumanii* are highlighted as critical pathogens [12]. The increased prevalence of CRE infections is a major threat to cancer patients [13]. Multiple studies demonstrate the impact of antibiotic resistance on outcomes in this vulnerable population. A study from Italy demonstrated 54% mortality due to CRE *Klebsiella pneumoniae* in neutropenic stem cell transplant patients [14]. Similarly a study by Satlin *et al*. demonstrated >50% mortality due to BSI with CRE *K. pneumoniae* in neutropenic patients [15].

Alert to the crisis of AMR, the World Health Assembly in May 2015 adopted a global action plan on antimicrobial resistance. One of the five strategic objectives is to strengthen knowledge through surveillance and research. Particularly important gaps in knowledge that need to be filled include the following: “information on the incidence, prevalence, range across pathogens, and geographical patterns related to antimicrobial resistance should be made accessible in a timely manner to guide the treatment of patients; to inform local, national, and regional actions; and to monitor the effectiveness of interventions” [16].

In the above context, this study aimed to evaluate the spectrum of bacterial pathogens and their antimicrobial susceptibility pattern in cancer patients with BSI at a tertiary care cancer hospital in central India over 5 years.

The findings will help formulate the antibiotic policy at the local level. In a broader context, it will be representative of the pattern of antimicrobial-resistant pathogens encountered in BSI in oncology patients from central India. The availability of updated data from various regions will help policymakers at the regional and national level to update the empirical antibiotic therapy options for cancer patients.

## Methods

### Study method

This single-centre retrospective observational study was conducted in a 250-bed cancer hospital that offers tertiary care in central India. The study included patients, both adult and paediatric, with solid organ and haematological malignancies whose blood cultures were received in the microbiology laboratory between January 2018 and December 2022. Blood cultures that were reported to have contaminants were excluded.

The study was approved by the institutional ethics committee of the National Cancer Institute (ref. no. NCI/EC/2018/039/01/2023; NCIEC reg. no. ECR/1130/Inst/MH/2018/RR-21).

### Microbiological processing

Two sets of blood cultures, aerobic and anaerobic (BACT/ALERT culture media), were collected aseptically from adult patients suspected of BSI. Similarly, blood cultures for paediatric patients were collected in paediatric blood culture bottles. The samples were immediately transported to the clinical microbiology laboratory and loaded in the BACT/ALERT (Durham, NC, USA) automated blood culture system.

A Gram stain slide was prepared from positive blood culture bottles. Blood culture bottles were subcultured on blood agar (Himedia) and MacConkey agar plates (Himedia) if the slides showed Gram-negative bacilli or Gram-positive cocci. The blood cultures showing budding yeast cells or fungal hyphae were subcultured on Sabouraud dextrose agar (SDA) from Himedia for fungal isolates. Bacterial identification and yeast identification to species level and AST were performed according to the recommendations of the Clinical and Laboratory Standards Institute (CLSI) using the automated identification and AST system (Vitek 2 compact system, bioMérieux, France) [17]. Filamentous fungi were identified morphologically by growth characteristics and preparing lactophenol cotton blue (LPCB) preparation from fungal growth on SDA. One *Paecilomyces* species isolate was sent to the Centre of Advanced Research in Medical Mycology and WHO Collaborating Centre, Department of Medical Microbiology, Post Graduate Institute of Medical Education and Research, Chandigarh, India for further identification and was identified as *Paecilomyces variotii*.

The following antibiotics were tested for Gram negative bacilli (Vitek R 2 AST-N280, Vitek R 2 AST- N 281), Gram positive bacilli (Vitek R 2 AST-P628) and Yeast (Vitek R 2 AST-YS08) (Tables1^3^).

**Table 1.** Antibiotic panel for Gram-negative bacilli

Gram-negative bacilli panel		
Class	Antibiotic	MIC Calling Range
Aminopenicillin/inhibitor combinations	Ampicillin	2–32
	Ampicillin/sulbactam	2/1 – 32/16
	Amoxicillin/clavulanic Acid	2/1 – 32/16
Ureidopenicillin / inhibitor combinations	Piperacillin/tazobactam	4/4 – 128/4
	Ticarcillin/clavulanic Acid	8/2 – 128/2
β-lactam /	Ceftazidime/avibactam	0.12–16
inhibitor combinations	Ceftolozane/tazobactam	0.25–32
	Meropenem/vaborbactam	0.5–64
Cephalosporin I	Cefalotin	2–64
	Cefazolin	4–64
Cephalosporin II/cephamycin	Cefotetan	4–64
	Cefoxitin	4–64
	Cefuroxime	1–64
	Cefotaxime	1–64
	Cefotaxime (reformulated)	0.25–64
	Cefpodoxime	0.25–8
	Ceftazidime	1–64
Cephalosporin III/IV	Ceftriaxone	1–64
	Ceftriaxone (reformulated)	0.25–64
	Cefepime	1–64
	Cefepime (reformulated)	0.12–32
Monobactam	Aztreonam	1–64
ESBL	ESBL confirmation test	+/–
	Ertapenem	0.5–8
	Ertapenem (reformulated)	0.12–8
Carbapenem	Imipenem	0.25–16
	Imipenem (reformulated)	0.25–16
	Meropenem	0.25–16
	Amikacin	2–64
	Amikacin (reformulated)	1–64
	Gentamicin	1–16
Aminoglycoside	Gentamicin (reformulated)	1–16
	Tobramycin	1–16
	Tobramycin (reformulated)	1–16
	Ciprofloxacin	0.25–4
Fluoroquinolone	Ciprofloxacin (reformulated)	0.06–4
	Levofloxacin	0.12–8
	Moxifloxacin	0.25–8
	Doxycycline	0.5–16
	Minocycline	0.5–32
Tetracycline	Tetracycline	1–16
	Tigecycline	0.5–8
	Tigecycline (reformulated)	0.5–8
	Nalidixic acid	2–32
Miscellaneous	Nitrofurantoin	16–512
	Trimethoprim/sulfamethoxazole	20(1/19)–320(16/304)

**Table 2.** Antibiotic panel for Gram-positive bacteria

**Gram-positive panel**		
Antibiotic	MIC Calling Range	FDA Indications for Use
Ampicillin		
*Enterococcus* spp.	2–32	*Enterococcus* spp.
*S. agalactiae*	0.25–16	*S. agalactiae*
Ampicillin/sulbactam	2/1–32/16	*Staphylococcus* spp.
Benzylpenicillin		
*Enterococcus* spp.	0.12–64	*Enterococcus* spp.
*Staphylococcus* spp.	0.03–0.5	*Staphylococcus* spp.
*Staphylococcus* spp.	0.03–0.5	*Staphylococcus* spp.
*S. agalactiae*	0.12–64	*S. agalactiae*
Cefoxitin screen	NEG–POS	*Staphylococcus* spp.
Ceftaroline	0.06–4	MRSA, MSSA
Chloramphenicol	4–64	*Enterococcus* spp., *Staphylococcus* spp.,
		*S. agalactiae*
Ciprofloxacin	0.5–8	*Enterococcus* spp., *Staphylococcus* spp.
Clindamycin	0.25–8	*Staphylococcus* spp., *S. agalactiae*
Clindamycin	0.125–4	MSSA, MSSE
Daptomycin	0.12–8	*S. aureus*, *VancomycinSusceptible E. faecalis*
Doxycycline	0.5–16	*S. aureus*
Erythromycin	0.25–8	*Enterococcus* spp., *Staphylococcus* spp.,
		*S. agalactiae*
Gentamicin	0.5–16	*Staphylococcus* spp.
Gentamicin high level (synergy)	S–R	*Enterococcus* spp.
Inducible clindamycin resistance	NEG–POS	*Staphylococcus* spp.
Levofloxacin	0.12–8	*Enterococcus* spp., *Staphylococcus* spp.,
		*S. agalactiae*
Linezolid	0.5–8	*E. faecium*, *E. faecalis*, *S. agalactiae*, *S. aureus*
		*S. epidermidis*, *S. haemolyticus*
Minocycline	0.5–16	*Enterococcus* spp., *Staphylococcus* spp.
Moxifloxacin	0.25–8	MSSA
Nitrofurantoin	16–512	*Enterococcus* spp., *Staphylococcus* spp.
Norfloxacin	0.25–16	*Enterococcus* spp., *Staphylococcus* spp.
Oxacillin	0.25–4	*Staphylococcus* spp.
Quinupristin/Dalfopristin	0.25–16	*MSSA*, *S. agalactiae*, *S. epidermidis*, *VancomycinResistantE. faecium*
Rifampicin	0.5–32	*Staphylococcus* spp.
Streptomycin high level (synergy)	S–R	*Enterococcus* spp.
Tetracycline	1–16	*Enterococcus* spp., *Staphylococcus* spp., *S. agalactiae*
		*E. casseliflavus*, *E. faecalis*, *E. faecium*
Tigecycline	0.12–2	*S. agalactiae*, *S. aureus*, *S. epidermidis*
		*S. haemolyticus*
Trimethoprim/sulfamethoxazole	10 (0.5/9.5)	*S. aureus*
	320 (16/304)	
Vancomycin	0.5–32	*Enterococcus* spp., *Staphylococcus* spp.,
		*S. agalactiae*

**Table 3.** Antifungal panel

Antifungal	MIC calling range	FDA indications for use
Caspofungin	0.125–8	*C. albicans*, *C. glabrata*, *C. guilliermondii*
		*C. krusei*, *C. parapsilosis*, *C. tropicalis*
		*C. albicans*, *C. dubliniensis*
Fluconazole	0.5–64	*C. guilliermondii*, *C. lusitania*
		*C. parapsilosis*, *C. tropicalis*
		*C. albicans*, *C. dubliniensis*, *C. glabrata*
Flucytosine	1–64	*C. guilliermondii*, *C. lusitaniae*
		*C. parapsilosis*, *C. tropicalis*
Micafungin	0.06–8	*C. albicans*, *C. glabrata*, *C. guilliermondii*
		*C. krusei*, *C. parapsilosis*, *C. tropicalis*
Voriconazole	0.12–8	*C. albicans*, *C. guilliermondii*, *C. krusei*
		*C. lusitaniae*, *C. parapsilosis*, *C. tropicalis*

### Data collection

The demographic data for the patients, including age, gender, and type of malignancy, were retrieved from their electronic medical records. The culture reports were retrieved from the microbiology laboratory data. Microbiological data, including the isolated pathogen IDs and their susceptibility patterns to antibiotics, were recorded. The epidemiology of the isolated pathogens and their susceptibility patterns recorded from blood cultures were evaluated. The demographics of the patients, including name, age, sex, diagnosis, microbiological pathogens isolated from blood cultures and their antibiotic susceptibility results, were documented in Microsoft Excel (RRID: SCR_016137).

### Statistical analysis

Data were entered and tabulated using Microsoft Excel (RRID: SCR_016137), and statistical analysis was performed using Statistical Package for Social Sciences (SPSS) version 29.0 (RRID: SCR_002865). The trend of antimicrobial resistance was evaluated using regression analysis. A *P*-value of <0.05 was considered significant.

## Results

### General characteristics

**Fig. 1.**
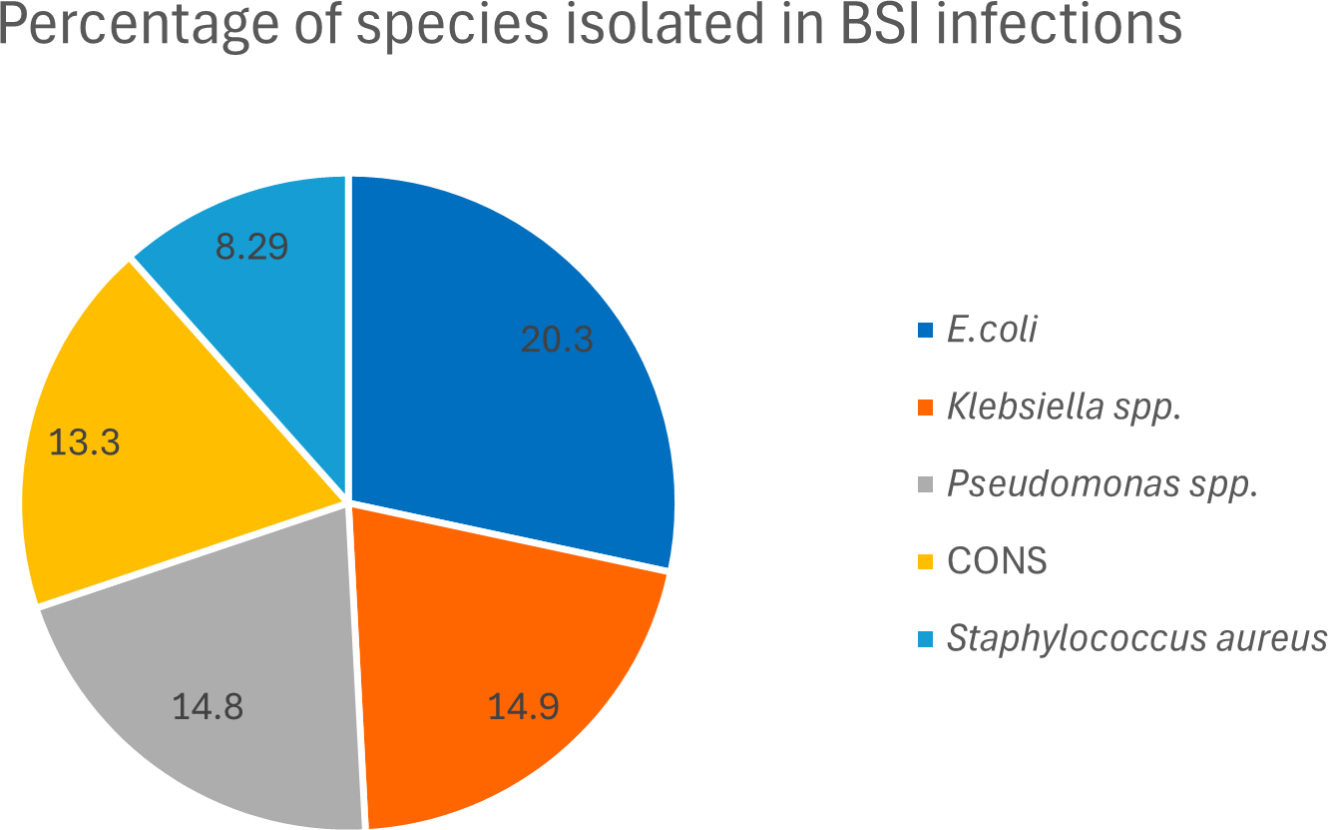
Percentage of common species isolated in BSI.

A total of 687 episodes of BSI in 524 patients were recorded. Among these patients, 59% were male, 68% were adult patients and 32% were paediatric patients; 63% of the patients were with haematological malignancy. Among the 687 isolates, 439 (64%) were Gram-negative bacilli (GNB), 175 (25%) were Gram-positive cocci (GPC), and 63 (9%) were fungal isolates. Ten cases were polymicrobial. The distribution of these pathogens and the commonest species causing BSI are presented in Table 4 and Fig. 1.

**Table 4.** Distribution of pathogens causing BSI

Microorganisms	No. (N)	Percentage (%)
Gram-negative micro-organisms	439	64
*Escherichia coli*	140	32
*Klebsiella* spp.	103	23
*Pseudomonas* spp.	102	23
*Acinetobacter* spp.	15	3
*Stenotrophomonas* spp.	13	3
*Sphingomonas* spp	13	3
Non-fermenter group	12	3
*Burkholderia* spp	11	2.5
*Serratia* spp	10	2
*Enterobacter* spp.	10	2
*Citrobacter* spp.	4	0.9
*Proteus* spp.	4	0.9
*Morganella* spp.	2	0.4
Gram positive micro-organisms	175	25
Coagulase-negative staphylococci	92	56
*Staphyloccus aureus*	57	36
*Enterococcus* spp.	20	11
*Streptococcus* spp.	6	3
Fungal micro-organisms	63	9
*Candida albicans*	19	24
*Candida* non-*albicans* spp.	42	73
*Paecilomyces variotii*	1	1.5
*Trichosporon*	1	1.5
Polymicrobial	10	1.5

### Distribution of pathogens (Table 4)

Among the total 687 pathogens, *Escherichia coli* (*n*=140) was the commonest isolated pathogen, followed by *Klebsiella* species (*n*=103), *Pseudomonas* species (*n*=102), and coagulase-negative staphylococci (*n*=92) (Fig. 1) Among 439 Gram-negative bacteria, *E. coli* was the most common pathogen isolated (32%), followed by *Klebsiella* species and *Pseudomonas* species (23% each).

Coagulase-negative staphylococci (CONS), which comprised 56% of all Gram-positive isolates, were the most frequently isolated, followed by *Staphylococcus aureus* isolates, which made up 36%. There were 20 enterococci species isolates (11%) and 6 streptococci species isolates (3%).

Among the 63 fungal isolates, *Candida* species were the most isolated. The proportion of non-*albicans Candida* species was high (72.5%) as compared to *Candida albicans* (24%). There were single isolates of *Paecilomyces variotii* and *Trichosporon*.

**Fig. 2.**
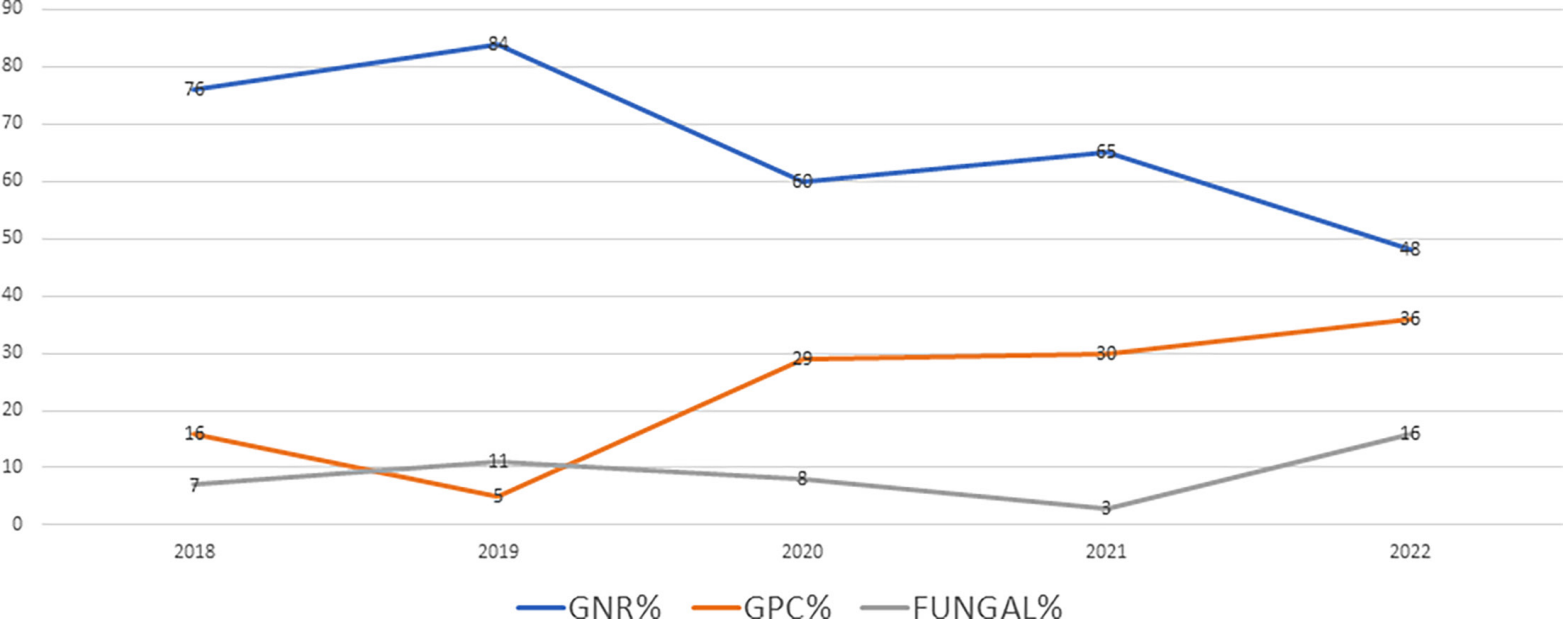
Trends for isolated pathogens over 5 years.

Over the 5 year study period, the trend of isolated pathogens (*P*=0.79 for GNR, *P*=0.07 for GPC and *P=*0.278 for fungal isolates) remained stable (Fig. 2). Gram-negative bacteria were the predominant pathogens isolated from BSI in this set of patients.

### Antimicrobial susceptibility pattern for predominant pathogens

*E. coli, Klebsiella* species and *Pseudomonas* species were most commonly isolated Gram-negative bacilli.

Among 140 isolates of *E.coli*, 66% (*n*=92) were extended-spectrum β-lactamase (ESBL) producers and 26% (*n*=36) were resistant to carbapenem. In 103 isolated *Klebsiella* species, 50% (*n*=52) were carbapenem resistant and 36% (*n*=37) were ESBL producers. Among enterobacterales (*E. coli* and *Klebsiella* species), the ESBL rate was 53% and the CRE rate was 34% in our study.

Carbapenem resistance was seen in 25% of *Pseudomonas* species and 53% of *Acinetobacter* species isolates.

No colistin resistance was noted for the above-mentioned species (Table 5).

**Table 5.** Antimicrobial resistance profile of common isolates

Gram-negative micro-organisms	ESBL	Carbapenem resistance	Colistin resistance
*Escherichia coli* (*n*=140)	92 (66 %)	36 (26 %)	0
*Klebsiella* spp.(*n*=103)	37 (36 %)	52 (50 %)	0
*Pseudomonas* spp. (*n*=102)	n/a	25 (25 %)	0
*Acinetobacter* spp.(*n*=15)	n/a	8 (53 %)	0
**Gram-positive micro-organisms**	**Methicillin resistance**	**Vancomycin resistance**	–
*Coagulase negative staphylococci*	60 %	0	–
*Staphylococcus aureus*	64 %	0	–
*Enterococcus* species	n/a	4 %	–

**Fig. 3.**
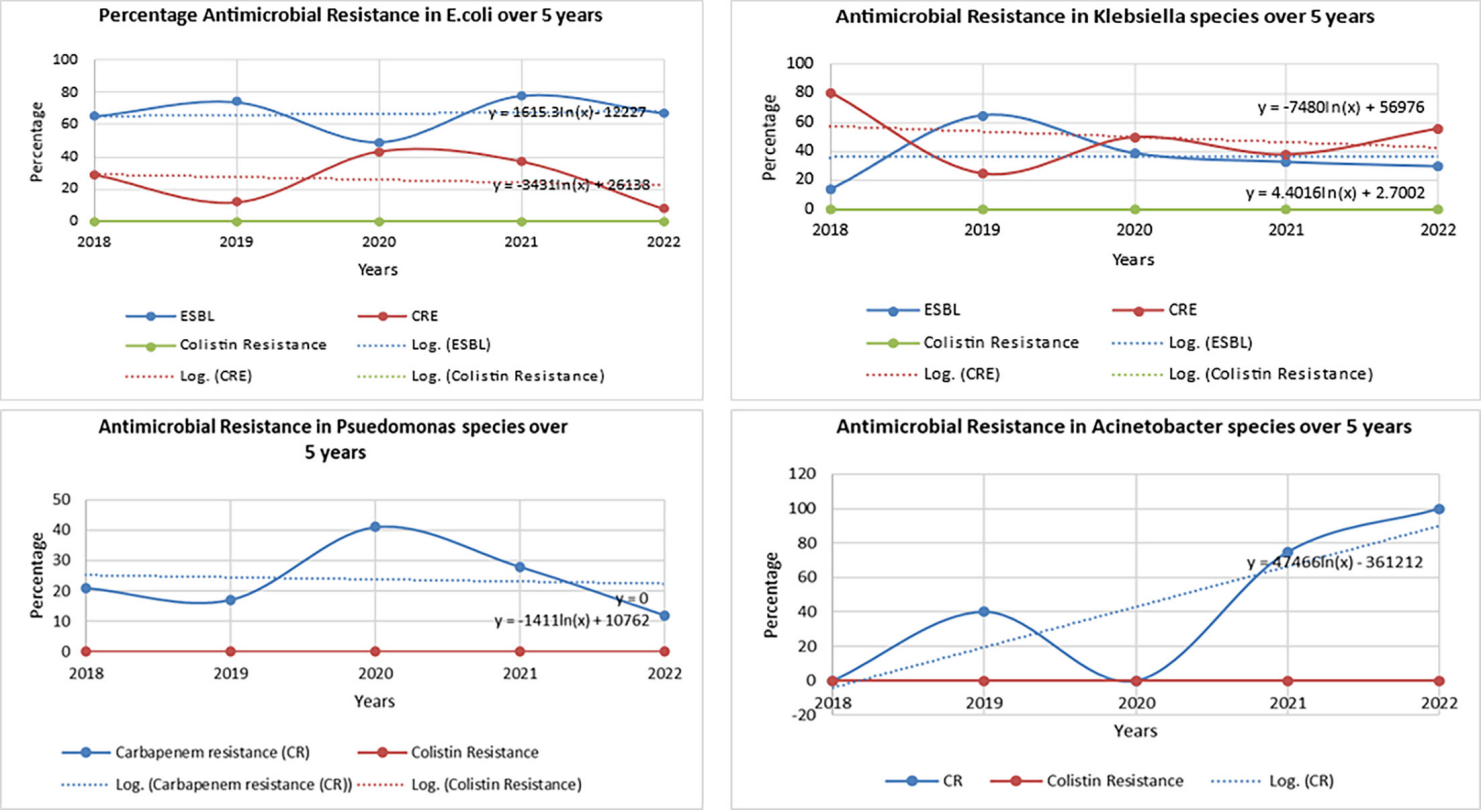
Trends of antimicrobial resistance in Gram-negative bacilli over 5 years from BSI. ESBL, Extended -Spectrum Beta-Lactamases; CRE, Carbapenem-Resistant Enterobacteriaceae.

Fig. 3 depicts the trends of antimicrobial resistance in Gram-negative bacilli over 5 years. There were no significant changes in the trends for ESBL *E. coli* (*P=*0.85), CRE *E. coli* (*P=*0.77), ESBL *Klebsiella* species (*P=*1) and CRE *Klebsiella* species (*P=*0.65) rates. Similarly, the trend for carbapenem resistance was insignificant for *Pseudomonas* species (*P=*0.87) and *Acinetobacter* species (*P=*0.08).

The overall sensitivity to third-generation cephalosporins was <40% in Gram-negative enterobacterales (Table 6). This may be due to a high prevalence of ESBL producers. The fourth-generation cephalosporin (i.e. cefepime) sensitivity was also ~30%. Similarly, <30% sensitivity was recorded for ciprofloxacin and trimethoprim/sulfamethoxazole in enterobacterales. The sensitivity to beta-lactam/beta-lactamase inhibitor (BL/BLI) drugs such as piperacillin/tazobactam, remained on the lower side, with *E. coli* exhibiting 53% sensitivity to it and *Klebsiella* species retaining only 37% sensitivity. Sensitivity to aminoglycoside ranged from 60–80% for *E. coli*, while it was <60% for *Klebsiella* species. Sensitivity to meropenem was 75% for *E. coli*, while it was only 50% for *Klebsiella* species*. Klebsiella* species showed higher resistance to all classes of antibiotics, except colistin and they were the most resistant pathogens.

**Table 6.** Percentage antibiotic susceptibility of predominant Gram-negative bacterial isolates

Organism	No of strains					% Susceptibility							
		Penicillin		Cephalosporins		Beta-Lactam/Bet	Lactamaseinhibitor	Aminoglycosides		Fluorpquinolones	Folatepathwayinhibitor	Carbapene ms	Polymixin
		Ampi-cillin	Cefuro-xime	Ceftria-xone	Cefepime	Amoxyclavula nicacid	Piperacillin-tazobactam	Amikacin	Gentamicin	Ciprofloxacin	TrimethoprimSulfamethoxazole	Meropenem	Colistin
*E. coli*	140	13	14	22	33	26	53	79	60	27	25	75	100
*Klebsiella* species	103	IR	19	22	31	22	37	54	53	28	29	50	100
*P.aeruginos a*	102	IR	IR	IR	62	IR	72	76	73	79	IR	75	100
*Acinetobac ter* species	15	IR	46	33	40	IR	47	47	53	40	40	67	100

Interestingly, *Pseudomonas* species retained sensitivity of >70% to piperacillin/tazobactam, aminoglycosides, ciprofloxacin, and meropenem in our set-up. *Acinetobacter* species exhibited 40–50% sensitivity to cephalosporins, piperacillin/tazobactam, aminoglycosides, ciprofloxacin, and trimethoprim/sulfamethoxazole, and 67% sensitivity to meropenem.

All *E. coli *and *Klebsiella*, *Pseudomonas*, and *Acinetobacter* species isolates were 100% sensitive to colistin.

Table 7 demonstrates that no resistance to vancomycin and linezolid was encountered in CONS and *S. aureus*. Sensitivity to teicoplanin was >80% for staphylococcal species and trimethoprim/sulfamethoxazole sensitivity was only 40–50%. Methicillin resistance was 60% in CONS and 64% in *S. aureus* (Table 5). One vancomycin-resistant enterococcus was isolated, leading to 96% sensitivity to vancomycin in enterococci. Linezolid sensitivity was 93% in enterococcus species*.* A resistance rate of >80% to ampicillin was encountered in enterococci.

**Table 7.** Percentage of Antibiotic Susceptibility of Predominant Gram-positive Bacterial Isolates

Organism	No of strains					% Suscept-ibility						
		Penicillin		Lincosami de	Macrolide	Folate pathway inhibitor	Glycopept ides	Oxazolidi nones	Aminoglyc osides	High-levelgentamicin(HLG)	Fluoroqui nolones	Lipoglyco peptides
		Oxacillin	Ampicillin	Clindamycin	Erythromyci n	Trimethopr m sulfamethox azole	Vancomycin	Linezolid	Gentamicin		Ciprofloxa cin	Teicoplanin
CONS	92	40	–	45	26	40	100	100	78	–	39	82
*S.aureus*	57	36	–	64	43	49	100	100	72	–	28	91
*Enterococ cus* spp.	20	–	26	IR	–	IR	96	93	–	67	37	–

Non-*albicans Candida* was the most common fungal pathogen. The sensitivity to fluconazole was 84% in non-*albicans Candida* species, while only one isolate of *C. albicans* was resistant to fluconazole.

## Discussion

This study provides information on local epidemiology of micro-organisms causing BSI and their antimicrobial susceptibility pattern in a set of immunocompromised patients from India.

Patients with haematological malignancies are more likely to develop BSI than those with solid organ cancers. The immunosuppression and mucosal barrier damage caused by the intense chemotherapy make these patients more susceptible to sepsis [18]. In addition, patients with haematological malignancies had a greater rate of blood culture positivity. Similar findings were reported from a study conducted at a tertiary cancer centre in Ghana [19,20].

In our set of patients, Gram-negative (GN) pathogens (64%) were most isolated from BSI followed by Gram-positive (GP) pathogens (25%) and fungal pathogens (9%). This aligns with the higher incidence of Gram-negative infections in a study conducted by Bhat *et al.* in oncology patients in India [21]. *E. coli* (32%), followed by *Klebsiella* species (23%) and *Pseudomonas* species (23%), were the most common GN pathogens isolated in this study. Similarly, in a study conducted by Perdikouri *et al.* in Greece, multidrug-resistant *Klebsiella* species and *Acinetobacter* species were the common pathogens in their cohort of cancer patients [22]. However, as opposed to our findings, Worku *et al.* from Ethiopia reported Gram-positive bacterial isolates, especially * S. aureus* as the most common pathogen in BSI in cancer patients from their centre [23]. The epidemiology of causative organisms causing BSI in cancer patients has changed over time. Regional variations among countries are also noted. An increasing incidence of multidrug-resistant Gram-negative bacteria has been reported in most studies in recent years [24,25]. The predominance of Gram-negative bacteria over Gram-positive bacteria may be attributed to several factors, such as improvement in the handling of venous catheters and chemotherapy associated with higher intestinal toxicity resulting in endogenous bacteraemia.

Among the Gram-positive isolates, CONS (56 %), followed by *S. aureus* (36%), remained the most common GP pathogens causing BSI in cancer patients at our centre. A study from Iran reiterates these findings [26]. A cross-sectional study from the USA shows equal distribution of GN and GP isolates in haematological malignancies with febrile neutropenia (FN) [27]. CONS bacteraemia is mainly related to foreign body-associated procedures such as intravenous catheters and an immunocompromised state in patients [28]. In contrast to the findings of our study, CONS have been reported as the most commonly isolated pathogen in blood cultures of febrile neutropenic patients by Kara Ali *et al.* from Turkey [29].

There was no significant change in the trends for isolated pathogens from BSI over 5 years, with Gram-negative bacterial infections being the predominant isolates in our study. As opposed to our trend, the study by Kara Ali *et al.* from Turkey concerning an 11-year cohort of febrile neutropenic patients showed a predominance of Gram-negative bacteria in blood cultures in 2011 and 2014, but with Gram-positive bacteria becoming more common in recent years [29]. A study from France concerning paediatric oncology patients showed no statistically significant change in BSI pathogens from 2019 to 2021 with staphylococcal BSIs being common in their set-up; however, *Enterobacteriaceae* BSIs gradually increased over 4 years [30]. The authors attributed gut colonization with multidrug-resistant bacteria and chemotherapy-induced mucositis and translocation as a cause of the rise in multidrug-resistant bacterial BSI.

Infections due to multidrug-resistant organisms (MDROs) are a major concern in cancer patients. Gram-negative bacteria showed a higher multidrug resistance pattern. Extended-spectrum beta-lactamase (ESBL) was observed in 53% in enterobacterales. Sevitha *et al.* reported 50.4% of ESBLs in *E. coli* and *Klebsiella* isolates [21] . Our centre has a carbapenem resistance rate of 34%, which is comparable to the prevalence observed in cancer patients in eastern India [31]. As stated above, the study from Greece reported 37% carbapenem-resistant *K. pneumoniae* and 21% carbapenem-resistant *Acinetobacter baumanii* out of 73 patients with mutidrug-resistant infections[22]. Colistin sensitivity was retained in 100% of enterobacterales in our study.

Sensitivity among third-generation and fourth-generation cephalosporins was 20–30% in *E. coli* and *Klebsiella* species. The sensitivity of enterobacterales to BL/BLI antibiotics (i.e. piperacillin/tazobactam) was very low (53% for *E. coli* and just 37% for *Klebsiella* species) (Table 6). Also noteworthy is a higher resistance (around 70%) rate to ciprofloxacin and trimethoprim/sulfamethoxazole in enterobacterales. A similarly high level of resistance was noted in a study from Sudan [28].

*Acinetobacter* species also showed a high level of resistance (50–60%) to cephalosporins, piperacillin/tazobactam, aminoglycosides, ciprofloxacin, and trimethoprim/sulfamethoxazole and a lower level of resistance (33%) to meropenem. Surprisingly, the *Pseudomonas* isolates retained sensitivity of 60–70% to cefepime, piperacillin/tazobactam, aminoglycosides, and ciprofloxacin, and 75% sensitivity to meropenem (Table 6). *Pseudomonas* and *Acinetobacter* species have emerged as the predominant multidrug-resistant bacteria globally. In a multicentre study conducted in Italy, 71% of these were multidrug-resistant. In a study by Ritvan Kara Ali *et al.*, 26.5% of *Pseudomonas* and 60.7% of *Acinetobacter* species had carbapenemase activity [29,32]. As opposed to this study, no colistin resistance was noted in our study for *Pseudomonas* and *Acinetobacter* species.

Among the staphylococcal bacteraemia cases, MRCONS were 64% and MRSA were 60%. One VRE isolate was reported in 5 years. No resistance to vancomycin and linezolid was reported for Gram-positive isolates. Staphylococcal isolates showed <60% sensitivity to erythromycin, trimethoprim/sulfamethoxazole, and ciprofloxacin, and >70% sensitivity was retained to gentamicin. The study from Sudan also reported similar findings with no vancomycin and glycopeptide resistance in GPC with higher resistance to erythromycin and tetracycline.

Considering our data, piperacillin/tazobactam monotherapy is not recommended at our centre as the antibiotic of choice for empirical treatment of patients with high-risk of FN and BSI. International guidelines prescribe monotherapy with mainstay antibiotics such cefepime, piperacillin/tazobactam, or an antipseudomonal carbapenem (imipenem or meropenem) as an initial antibiotic regimen (IAR) for stable patients presenting with FN [33,35]. However, the epidemiological data used to make these antibiotic recommendations were collected approximately 20 years ago, before the onset of widespread Gram-negative antibiotic resistance [36,37]. Based on the data from this study, we recommend tailoring of antibiotic treatment according to the local epidemiology and prevalence of mutidrug-resistant organisms. A prospective multicentre study of BSI in febrile neutropenic patients from 2006–2017 also highlights that the increasing trend of multidrug-resistant Gram-negative bacilli in such patients is associated with inadequate empirical antibiotic therapy, in spite of adherence to IDSA guidelines [24].

After this study, we highlighted the importance of restricted use of higher antibiotics, use of targeted therapy based on microbiological confirmation, de-escalation practices and strengthening of infection control practices at our institute.

The study had certain limitations. It was a single-centre retrospective study. We did not study the risk factors for BSI and outcomes associated with it, in this set of patients.

The strength of this study is that it included large number of vulnerable patients over 5 years. This substantial data have good generalizability for application in cancer patients.

## Conclusion

Recent microbial patterns in oncological patients have shifted towards multidrug-resistant (MDR) strains; this can harm patient outcomes. Therefore, it is crucial to assess the risk of MDR infections in every cancer patient. Prompt initiation of effective antibiotics can be beneficial for patient outcomes. There is a significant need for increased surveillance of MDR bacteria, implementation of effective antimicrobial regimens combined with antibiotic stewardship programmes, and wide adoption of infection control measures in oncology departments. This can be ensured by accessibility to robust Clinical Microbiology diagnostic services, continued surveillance of epidemiology of pathogens and antimicrobial resistance in such vulnerable patients, and risk assessment for MDR infections in cancer patients.

## Abbreviations

AMR: antimicrobial resistance
AST: antimicrobial susceptibility testing
BSI: blood-stream infection
CONS: coagulase-negative *Staphylococci*
CRE: carbapenam resistant enterobacterales
ESBLs: extended-spectrum beta lactamases
IDSA: Infectious Diseases Society of America
LPCB: lacto-phenol cotton blue
MDROs: multi-drug resistant organisms
SDA: Sabouraud's dextrose agar
WHO: World Health Organisation
